# Are Public Oral Care Services Evenly Distributed?—Nation-Wide Assessment of the Provision of Oral Care in Japan Using the National Database of Health Insurance Claims

**DOI:** 10.3390/ijerph182010850

**Published:** 2021-10-15

**Authors:** Tomoko Kodama, Yusuke Ida, Katsuo Oshima, Hiroko Miura

**Affiliations:** 1 Department of International Health and Collaboration, National Institute of Public Health, Saitama 351-0197, Japan; 2 Healthcare Executive Program, Graduate School of Medicine, The University of Tokyo, Tokyo 113-8655, Japan; yskida@m.u-tokyo.ac.jp; 3 Department of Dental Technology, The Nippon Dental University College, Tokyo 102-8159, Japan; oshima@tky.ndu.ac.jp; 4 Division of Disease Control and Epidemiology, School of Dentistry, Health Sciences University of Hokkaido, Tobetsu-cho 061-0293, Hokkaido, Japan; hmiura@hoku-iryo-u.ac.jp

**Keywords:** oral health, oral care services delivery, database of information on receipts and specified health examinations, regional differences, standardized claims data ratio

## Abstract

The provision of oral health care services is one of the global challenges under the realization of universal health coverage in many countries. Despite the increasing importance of oral health care in an aging society, the disparities in the provision of oral care in Japan have not been clarified. Therefore, this study investigated the status of oral and dental care provision using the National Database of Health Insurance Claims and Specific Health Checkups (NDB) at the level of prefectures and secondary medical care areas. Additionally, a multiple regression model was applied to identify the influence of human resources in oral care services and economic factors on the standardized claims data ratio (SCR) of total dental receipts. The results showed that the total amount of oral care provided tended to be higher in metropolitan areas, with bimodal peaks in children aged 5–9 and adults in their 70s. The SCR for dental caries showed little difference nationally, but SCR for periodontal disease tended to be higher in prefectures including metropolitan areas. In a multiple regression model, the number of dentists and prefectural income per capita influenced the SCR of total dental receipts. In secondary medical care areas, some depopulated areas are supplemented by adjacent areas. These results suggest that oral health care services in the national health insurance system are generally well provided; however, they are likely to be influenced by human resources and economic disparities, and regional differences may occur in the care of periodontal diseases.

## 1. Introduction

In 2020, the WHO General Assembly adopted the achievement of better oral health as part of the Universal Health Coverage and Non-Communicable Diseases agenda for 2030 [1]. Oral diseases are prevalent worldwide and are known to be a serious health and economic burden, significantly reducing the quality of life [2]. Furthermore, it has been reported that poor oral health in older people is associated with a high risk of subsequent deterioration in general health [3]. For example, poor oral health has been reported to be associated with a higher risk of mortality from cardiovascular diseases (CVD) [4,5,6,7], diabetes [8], pneumonia mortality [9,10,11], and cancer [9,12].

According to the United Nations, the world’s population is aging rapidly, and people aged 65 years and over are expected to be one in six people worldwide by 2050 [13]. Therefore, the need for oral health care in conjunction with preventive care has recently gained importance [14]. Due to the aging population in Japan as well as other countries, oral and dental care services are undergoing a major transformation. Oral and dental care and treatment in Japan is covered by public national health insurance with a 30% co-payment (20% or even free for children depending on municipalities) as other general medical care and treatment. The co-payment of medical expenses for people aged 70 and over is 10–20%, depending on their income level [15]. Under the National Health System, a minimum amount of medical cost is guaranteed for people in need; however, public insurance only covers care or treatments approved by the board of the committee [16]. In some cases, such as metal dentures, people need to pay the difference between the cost of insurance and the cost of the self-covered treatment.

Therefore, in order to measure actual oral and dental care delivery in Japan, it is necessary to use actual medical receipts. The National Database of Health Insurance Claims and Specific Health Checkups of Japan (NDB) is a database consisting of receipts (medical fee statements) issued by medical institutions including dentistry to insurers when patients visit clinics and hospitals [17,18,19]. The main information contained in a receipt includes patient information such as name, date of birth, and gender; information on the medical institution, insurer, and insured person; as well as information related to medical treatment such as the name of the disease, treatment details, medication, and equipment used. However, not all data are stored in the NDB as it is. The patient’s name and date of birth, location and name of the insured medical institution, medical record number, and the insured person’s card number are removed so that the individual cannot be identified.

Previous reports using the NDB in dentistry indicated that those with fewer current teeth or more missing teeth were more likely to have received treatment for aspiration pneumonia at medical institutions among the older population [20]. Another study reported that regular visits to dental institutions from an early age, focusing on the prevention of dental diseases such as dental caries and periodontal disease, which are the main causes of tooth loss, may reduce not only dental care costs but also overall health care costs [21]. However, none of the previous reports mentioned the healthcare provision system in the whole country. Therefore, this study investigated the status of oral and dental health care provision at the prefectural level and secondary medical care in Japan using the NDB. In addition, since the amount of dental care provision is closely linked to the workloads of dentists [22] and other oral and dental care workers, such as dental hygienists and dental technicians [23,24], we examined the influence of human resources and prefectural economic indicators from national statistics on the provision of oral and dental care services.

## 2. Materials and Methods

### 2.1. Data Acquisition

In this study, we appealed to the Ministry of Health, Labour, and Welfare (MHLW) for the use of individual monthly oral and dental care receipts from April 2016 to March 2017 (FY2016) and April 2018 to March 2019 (FY2018) on 18 July 2019, according to NDB guidelines [25]. The data were provided to researchers in December 2019 following a review by the Advisory Committee on NDB use. The items of dental receipt information covered a number of dental diseases (three groups of major dental diseases: 89 codes related to dental caries, 51 codes related to periodontal disease, and 10 codes related to tooth loss) [26].

### 2.2. Data Analysis

#### 2.2.1. Description for Dental Claim Data

The number of age-specific dental claim data per 1000 people (standardized by the national population for 5-year age groups) by gender was calculated. The number of dental claim data in FY2016 and FY2018 were also calculated for all 47 prefectures in Japan and were categorized into 6 administrative regions, as follows: *Hokkaido Tohoku*, *Kanto*, *Chubu*, *Kansai*, *Chugoku Shikoku*, and *Kyushu Okinawa*, from north to south in order. A one-way analysis of variance (ANOVA) was conducted to examine regional differences in issuing receipts.

#### 2.2.2. Calculation of the Standardized Claims Data Ratio (SCR)

The Standardized Claims Data Ratio (SCR), adjusted for gender and age (5-year age groups) was used to measure the use of oral care services in 47 prefectures [27] to compare the amount of dental and oral care prescriptions, which is similar to the way the Standardized Mortality Ratio (SMR) compares mortality rates between regions with different age structures, as follows:

SCR = {Σ (number of receipts by age group)/Σ (expected number of receipts by age group)} × 100(1)

= {Σ (number of receipts by age group)/Σ (population by age group × national incidence of receipts by age group)} × 100(2)

First, we calculated the SCR for total dental receipts. Then, we calculated the SCRs for three disease categories—dental caries, periodontal diseases, and tooth loss—according to the codes defined by the Ministry of Health, Labor, and Welfare.

#### 2.2.3. Geographic Analysis Using Z-Score for Distribution of Oral and Dental Care Services in Secondary Medical Care Area

For the comparison of secondary medical care areas, the distribution was assessed using the Z-score for every thousand people. Because the sample size was limited for calculating the SCR of each secondary medical care area, the Z-score was used for comparison. The Z-score is a value transformed such that the mean is 0 and the standard deviation is 1, and it represents the variation from the mean. For these reference populations, we used 2016 and 2018 municipal population data from the Statistics Bureau of the Ministry of Internal Affairs and Communications.

The secondary medical care areas in Japan are defined by the Medical Care Act as regional units for the development of regional medical care to be provided (i.e., emergency medical care, medical care in times of disaster, perinatal care, remote area medical care) [28]. This unit refers patients from clinics (outpatients) to hospitals to receive secondary care. Although the number is very small, there are hospitals offering dental care and treatment for outpatients at hospitals, and we also calculated the total number of visits for both (outpatient) hospitals and clinics.

ArcGIS Pro 2.3.3 (ESRI) (Environmental Systems Research Institute. Redlands, CA, USA) was used as the geographic analysis software, and visualization was performed by mapping to secondary health area codes. Graphic information from the administrative area 2.3 version of the National Land Information and Administrative Area Data was used for the prefecture-wise analysis, and the medical area data version 1.0 were used for the analysis by secondary medical area.

#### 2.2.4. Associations with the Prefectural Characteristics of Human Resources and Economic Indicator

We analyzed the influence of the factors related to human resources and economic state at the prefecture level on the SCR of total dental receipt. We collected publicly available national statistics at the prefecture level, such as the number of dentists in practice at healthcare facilities [29], dental hygienists [30], dental technicians [30], dental laboratories [30], registered dental clinics in national health insurance [31], and income per capita in each prefecture (1000 JPY = USD 9.1 in August 2021) [32].

We used Spearman’s correlations to examine the associations between the SCR of total receipts and prefectural characteristics in the oral and dental care delivery system. A multiple linear regression model was applied to assess the strength of the relationship between the SCR of total receipts as an outcome variable and prefectural characteristics as predictor variables. The predictor variables were the number (per 1000 people) of dentists, dental hygienists, dental technicians, dental laboratories, dental clinics, and prefectural income per capita (million JY).

In Model 1, the predictor variables were the number (per 1000 people) of dentists, dental technicians, and prefectural income per capita. In Model 2, the predictor variables were the number (per 1000 people) of dental clinics, dental laboratories, and prefectural income per capita. In Model 3, the number (per 1000 people) of dental hygienists was added to Model 1. The normality of the residuals was examined in each model using a quantile–quantile plot. All variables were independent, and the interaction was examined between variables of the number of dentists and prefectural income per capita in Models 1_a and 3_a, as well as for the number of clinics and prefectural income per capita in Model 2_a. The variables were centralized to avoid multicollinearity in the regression model.

For statistical analysis, STATA SE/16 (Stata Corp LP, College Station, TX, USA) was used. A two-tailed test was used with *p* < 0.05 considered statistically significant.

## 3. Results

### 3.1. Distribution of Dental Receipts by Age and Gender

**Figure 1:**
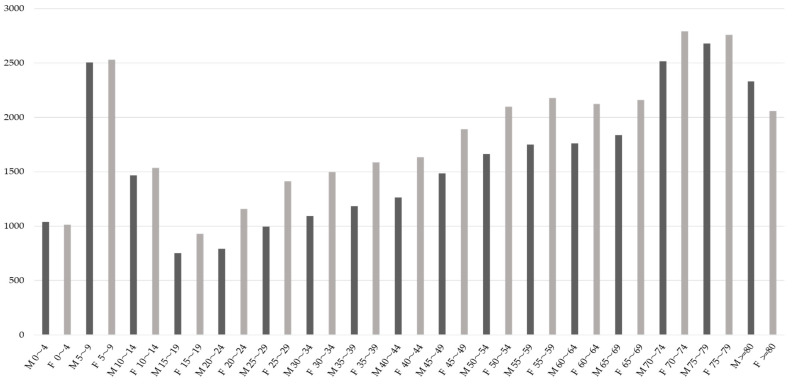
The number of age-specific dental receipts per 1000 people by gender in FY2018. M: male, F: female.

The number of dental claims data per 1000 people by gender and age group is shown in Figure 1. There was an initial peak between the ages of 5 and 9 years, the lowest between 15 and 19 years, then an increase again, and a second peak at 70 years. There was little difference between males and females under the age of nine, but females tended to be more prevalent in those aged 10 years and older. This trend was similarly observed in both FY2016 and FY2018.

### 3.2. Standardized Claim Data Ratio (SCR) in Oral and Dental Care Provision

**Figure 2:**
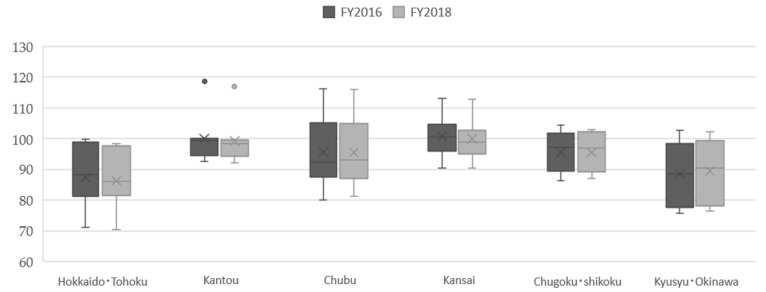
The Standardized Claim Data Ratios (SCRs) of total dental receipts by administrative regions in Japan, FY 2016 and 2018.

The SCRs of the total number of dental claims data are presented as box-and-whisker diagrams according to six administrative regions (Figure 2).

The six administrative regions are *Hokkaido* *Tohoku* (Hokkaido, Aomori, Iwate, Miyagi, Akita, Yamagata, Fukushima), *Kanto* (Ibaraki, Tochigi, Gunma, Saitama, Chiba, Tokyo, Kanagawa), *Chubu* (Niigata, Toyama, Ishikawa, Fukui, Yamanashi, Nagano, Gifu, Shizuoka, Aichi), *Kansai* (Mie, Shiga, Kyoto, Osaka, Hyogo, Nara, Wakayama), *Chugoku* *Shikoku* (Tottori, Shimane, Okayama, Hiroshima, Yamaguchi, Tokushima, Kagawa, Ehime, Kochi), and *Kyushu* *Okinawa* (Fukuoka, Saga, Nagasaki, Kumamoto, Oita, Miyazaki, Kagoshima, Okinawa).

Regarding the difference between prefectures, the national baseline was set at 100, as mentioned previously, and the highest SCR was found in Tokyo (118.5 in FY2016 and 120.7 in FY2018, *Kanto* region). In the northern part of Japan, the *Hokkaido* and *Tohoku* regions, the SCRs of total claim data were all below the standard 100, and a similar trend was observed in the southern part of Japan, the *Kyushu* and *Okinawa* regions. In other parts of Japan, in the *Kanto* region (including Japan’s capital Tokyo), the *Chubu* region (the central part of Japan), and the *Kansai* region (including Osaka, the second largest city after Tokyo), there was greater variation within these regions. There were little differences in prefectural SCR between fiscal years 2016 and 2018, with an average change of −0.23%. There was no remarkable difference between the six regions by one-way analysis of variance (ANOVA), with *p* = 0.0766 and R2 = 0.2382 in FY2016, and *p* = 0.0953 and R2 = 0.2268 in FY2018.

### 3.3. The SCRs by Disease Codes (Dental Caries, Periodontal Disease and Tooth Loss)

**Figure 3:**
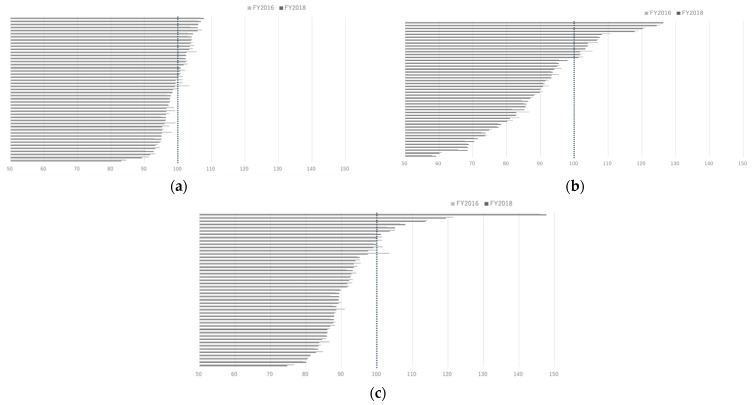
(**a**) The Standardized Claim Ratios (SCRs) of dental caries among 47 prefectures in FY2016 and FY2018. (**b**) The SCRs of periodontal disease among 47 prefectures in FY2016 and FY2018. (**c**) The SCRs of tooth loss among 47 prefectures in FY2016 and FY2018.

The SCRs calculated by classification into dental caries, periodontal disease, and tooth loss are shown in Figure 3.

The SCR for dental caries was found to have the least variation in the data. In contrast, SCRs for periodontal disease had a wide range of data and tended to be higher in prefectures with mega cities such as Tokyo, Osaka, and Nagoya. The SCR of lost teeth was the highest in Osaka Prefecture.

### 3.4. Z-Scores of Issued Dental Receipts in Secondary Medical Care Areas

**Figure 4:**
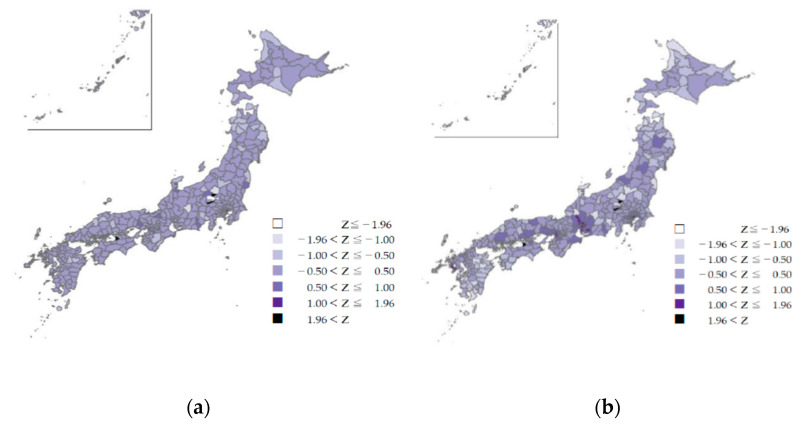

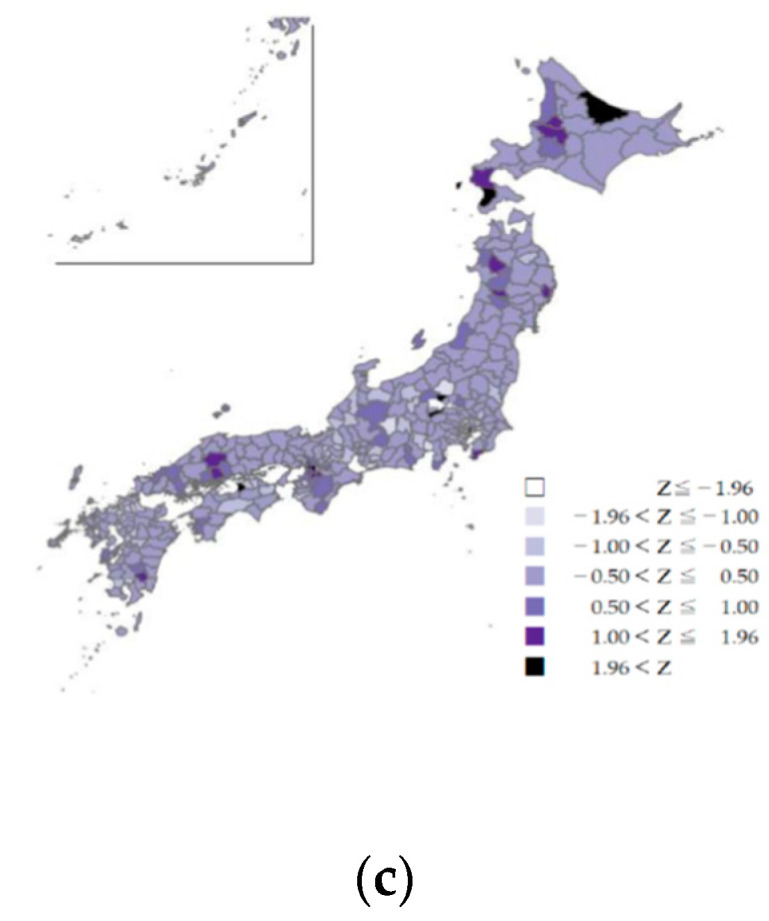
(**a**) The z-scores of dental caries by secondary medical areas in Japan, FY2018. (**b**) The z-scores of periodontal diseases by secondary medical areas in Japan, FY2018. (**c**) The z-scores of tooth loss by secondary medical areas in Japan, FY2018.

The Z-score per thousand people according to the secondary medical area code was higher than the average in urban areas. In urban areas (e.g., Tokyo and prefectural capitals), the number of receipts related to periodontal disease was higher than the national average, while the number of receipts related to dental caries and tooth loss tended to be lower (Figure 4a–c).

Except in mountainous and depopulated areas, the provision of national oral health care services for these three diseases was not remarkably low in the secondary medical areas. The areas in the center of the main Japanese island with z-values below −1.96 (colored white) are mountainous highlands with a small population. Adjacent areas had z-values higher than 1 or 1.96. This may be due to complementary medical resources in adjacent areas. A similar trend was observed in parts of Hokkaido in the SCR of tooth loss (northernmost part of the country), with z-values higher than 1.96.

### 3.5. Human Resources of Oral and Dental Care Services and Economic Indicator in Prefectures—Analysis for the SCR of Total Dental Receipt in Regression Model

Table 1 shows the prefectural characteristics of human resources of oral and dental care services and prefectural income per capita in FY2016 and FY2018.

The largest number of dental professionals per 1000 inhabitants in each prefecture were dental hygienists (1.06 in FY2016 and 1.13 in FY 2018), followed by dentists (0.73 and 0.74) and dental technicians (0.32 and 0.30). The number of dental hygienists was about 1.3 times that of dentists, while the number of dental technicians was about one-third that of dentists. The greatest disparity in the maximum over minimum comparison between the prefectures was found in the number of dental technicians per 1000 people, with a gap of almost four times. The others were relatively similar in the maximum over minimum comparison, which was almost double.

In Table 2, Spearman’s correlations show a positive association between SCR and the number of dentists (0.522, *p* < 0.001), dental clinics (0.501, *p* < 0.001), and prefectural income per capita (0.399, *p* < 0.001).

A negative correlation was found between SCR and dental technicians (−0.419, *p* < 0.05). There was also a positive relationship between the number of dentists and clinics (0.799, *p* < 0.001), the number of dentists and dental laboratories (0.376, *p* < 0.01), dental hygienists and dental technicians (0.399, *p* < 0.05), and dental clinics and dental laboratories (0.357, *p* < 0.05). Negative relationships were found between the number of dental hygienists and prefectural income per capita (−0.360, *p* < 0.05).

Table 3 shows the results of multiple linear regression analysis.

In Model 1, the number of dentists (Coef. 32.82, *p* = 0.001) and prefectural income per capita (Coef. 6.48, *p* = 0.02) were positive predictors, while the number of dental technicians had a negative influence (Coef. −21.86, *p* = 0.06). The model showed an improvement by adding an interaction term between the number of dentists and prefectural income per capita (squared R increased from 0.4658 to 0.5107).

In Model 2, dental clinics (Coef. 57.03, *p* = 0.011) and prefectural income per capita (Coef. 7.21, *p* = 0.02) were positive predictors. In Models 2 and 2_a, the addition of the interaction term between clinics and income reversed the slope of the coefficient for the dental laboratories, but the effect was not significant.

Model 3, which is a model of Model 1 with the addition of the number of dental hygienists, showed an improvement in goodness of fit over Model 1 (squared R increased from 0.4658 to 0.5296). Model 3_a is a model with an additional interaction term between dentists and prefectural income per capita (*p* = 0.036) and is the best-fitting model for the predictor variables considered here (R^2^ = 0.5799). The normality of the residuals was confirmed in all models.

### 3.6. Provided Oral Care at Hospitals and Clinics

In the category of total number of visits (outpatient), 97.2% of them were accounted for by clinics. Across all prefectures, clinics received an average of 19.9 times as many dental receipts as hospitals, indicating that clinics are by far the most common source of dental care services under national health insurance. In terms of the ratio of clinics to hospitals (per 1000 people) by prefecture, we found that Tokyo (*Kanto* region), Osaka (*Kansai* region), and Aichi (*Chubu* region) had more dental receipts from clinics than any other prefecture. We found fewer receipts from both hospitals and clinics in some prefectures in the *Tohoku* and *Kyushu* regions (data not shown).

## 4. Discussion

### 4.1. Standardized Claims Data Ratio (SCR) as an Indicator of Oral and Dental Care Provision

In this study, we used the SCR, which is a gender- and age-adjusted score developed by Fujimori et al. [27]. The concept is similar to the Standardized Mortality Ratio, which is the ratio of the number of claims observed in a population over a given period to the number expected over the same period. This index is based on the national average of healthcare provision (100), with a value above 100 indicating a high level of healthcare provision from the relevant receipts, and a value below 100 indicating a low level of healthcare provision. This score is used to identify the regional healthcare planning issues associated with medical services in Japan, because NDB is a complete dataset, which covers 96.4% in dentistry as of April 2018 [33].

The highest SCR was found in the capital, Tokyo, which was 1.7 times higher than those in the prefecture with the lowest SCR. The SCR tended to be lower in the northern and southern regions (*Tohoku, Hokkaido*, *Kyushu,* and *Okinawa*), which were distant from large cities such as Tokyo and Osaka. In our study, a comparison of projected and actual figures for total receipts categorized by age group showed that the volume of medical care provided to older people exceeded the national average in prefectures centered in Tokyo and Osaka, but was below the national average in prefectures with a lower income per capita within small populations and depopulated areas, such as in the north (Aomori, Wakayama), southwest (Tottori, Shimane), and south (Kagoshima, Okinawa). In fact, a significant positive correlation was found between prefectural income per capita and the SCR of total dental receipts, suggesting that the economic scale might be associated with the provision of oral and dental care, even under the National Health Insurance. This is also evidenced by the fact that in another study, the effect of health care expenditure on the prevalence of dental caries in early childhood was stronger than the effect of UHC service coverage [34]. Studies examining whether an individual’s economic resources, such as socio-economic status and dental insurance coverage, affect access to dental care have been reported in many countries [35,36,37,38,39,40]. In Korea, the prevalence of periodontal diseases decreased after the scaling coverage policy in health insurance came to existence [36].

### 4.2. Equitable Access to Oral and Dental Care

Receipt information quantifies the actual oral and dental treatments that are rendered. Regarding the SCR of three main diseases and conditions, such as dental caries, periodontal disease, and tooth loss, the SCR for dental caries was found to have the least variation in the data presented in our study. This was also proved by the mapping of the Z-scores. In remote mountainous areas, the areas with the highest and lowest Z-scores were adjacent to each other, suggesting that the provision of dental care is mutually supported in adjacent areas. This means that there is equitable access to caries treatment throughout Japan.

According to the 2016 Dental Diseases Survey Report, the prevalence of caries in deciduous teeth was 8.6% at the age of 3 years, increased from the age of 4 years, and was reported to be highest in children aged 9 years at 60% [41]. This increase in the prevalence of dental caries in children aged 5 to 9 years coincides with the peak in the number of receipts for children in the present analysis.

### 4.3. Oral and Dental Care Covered by Health Insurance

In Japan, the treatments covered by insurance are those that are necessary to cure a disease or to restore lost function. For example, implant treatment is not fully covered by health insurance, unlike treatments such as dental caries or periodontal disease. Implant treatment is only covered by public insurance if the patient’s jawbone is defected by treatment of malignant tumor or congenital diseases and the hospital’s equipment standards are met in a service delivery system with adequate staff, safety management, dentists with experience with such treatment procedures, etc. Therefore, at certain points, the national insurance system can lead to safer treatment options.

In our analysis using the regression model, SCR of total dental receipts was related to the number of dentists and clinics, but not to the number of dental technicians (negative relations) and dental laboratories. In Japan, dental technicians manufacture various types of dental prostheses under the orders of the dentist. Unlike dental technicians in some countries, the Japanese system prohibits dental technicians from undertaking activities that involve direct contact with the patient’s oral cavity. The main place of work for dental technicians is the dental laboratories. Most dental laboratories have only a few workers, but there are some dental laboratories that have a large number of workers, and they tend to be located in rural areas of western Japan [30]. For this reason, the number of dental technicians also tends to be higher in rural areas of western Japan, and is negatively correlated with dental clinics.

In regression analysis, the number of clinics and the number of dentists were modeled separately to ensure the independence of the variables. This is due to the fact that 89% of the dentists engaged in practice in Japan work in clinics [29].

In Japan, the largest number of dental professionals in FY2018 was dental hygienists (*n* = 132,629), followed by dentists (*n* = 101,777) and dental technicians (*n* = 34,469) (not shown in table). To evaluate the dental care system, it is necessary to examine the balance of various dental professionals. In the present study, the number of dental hygienists was not correlated with SCR, but was positively influenced in the multivariate models. The main tasks of dental hygienists are to assist dental care, to provide health guidance, and preventive care. The law states that dental hygienists must operate medical equipment or provide medicines under the supervision of a dentist; therefore, their services are limited under the current health insurance system. There was also a correlation between the number of dental hygienists and dental technicians, which may indicate that there is a greater need for both professionals in areas with fewer dentists, but the details could not be confirmed in this study.

### 4.4. Oral and Dental Care in Aging Societies

In this study, there was no clear trend in the proportion of the population aged 65 and over and the total number of receipts in our additional analysis, although it was higher in some medical areas such as Osaka (Miyakojima-ku, etc.), Aichi (Chikusa-ku, etc.), Tokyo (Chiyoda-ku, etc.), and Hokkaido (Chuo-ku, etc.).

The number of receipts for periodontal disease was higher in prefectures including metropolitan areas such as Tokyo and Osaka, as well as in other urban secondary care areas. There are two possible reasons for the wide variation in the SCRs of periodontal disease. First, the prevalence of periodontal disease may be higher and the number of patients may be increasing in those areas, and the second is that treatment of periodontal disease may be inadequate outside of urban areas. For the latter, this may be due to the higher awareness of prefectural residents in urban areas who may visit dental institutions for periodontal care.

Unlike dental caries, periodontal disease is often painless and can go unnoticed until it becomes severe, the gums bleed, and the tooth falls out spontaneously. Periodontal disease is a common cause of tooth loss, but it causes few symptoms, especially in the early stages. In Japan, the proportion of people with periodontal pockets according to the Community Periodontal Index increases with age, with the majority of people being over 45 years of age, and gingival bleeding is also present in about 40% of all age groups [41].

In recent years, the number of teeth at risk of developing periodontal disease has increased because of better tooth retention, especially in developed countries. In addition, it has been reported that periodontal disease is linked to systematic inflammation and is a risk factor for cardiovascular diseases, including stroke, diabetes, and other inflammatory diseases [42,43,44,45,46,47,48,49]. Therefore, oral health care professionals must embrace the concept of lifelong emphasis on prevention and participate as active members of the healthcare team providing care to the increasing older people in a variety of settings (hospital and clinic-based care, community-based settings, long-term care facilities, etc.), along with revisions to existing older adult insurance schemes [50].

As the population ages, it will be necessary to conduct future surveys on the situations of comprehensive community care, including oral and dental care. Furthermore, adequate medical and dental cooperation is needed in the community to improve individual health and chronic diseases.

### 4.5. Disparities in Oral Health Care—Global Issues

According to the Global Burden of Disease Study 2017, it is estimated that oral diseases affect nearly 3.5 billion people and an estimated 2.3 billion people suffer from caries of the permanent teeth and more than 530 million children suffer from caries of the primary teeth [51]. There is concern that low- and middle-income countries have a higher prevalence of oral diseases due to inadequate exposure to fluoride (found in tap water and oral hygiene products such as toothpaste) and inadequate access to oral health services in the community. In addition, uncontrolled marketing of sugary foods and beverages and tobacco and alcohol leads to increased consumption of products that contribute to oral diseases and other non-communicable diseases.

According to a survey of adults who have expressed a need for oral health services, access to oral health services is 35% in low-income countries, 60% in lower-middle-income countries, 75% in upper-middle-income countries, and 82% in high-income countries [52]. Insurance coverage for dental care is generally limited and accounts for 20% of all out-of-pocket expenditure across OECD countries [53]. In the Netherlands, it is estimated that on average, between 21% and 32% of oral care consumption is financed privately and data on total cost of the consumption from health insurers are limited to use [54]. Although we believe that a significant amount of money is spent on dental treatment in in Japan, it is difficult to know exactly how much individuals are paying for dental treatment that is not covered by insurance.

Disparities in oral health and access to dental care indicated that only 1 in 5 children covered by Medicaid received preventive oral care in the U.S. [55] and also those from low- and middle-income households in Korea were less likely to receive treatment than those from high-income households [56]. A comparative study between Japan and England reported that lower income and educational attainment were significantly associated with a higher risk of edentulism, and that Japan, with its wider coverage of dental care, had lower levels of inequality than England [57]. This study also supported that coverage of dental (oral) care was sufficient especially for dental caries.

In the United States, rural residents were reported to be less likely to receive diagnostic services (adjusted odds ratio (AOR): 0.82) and preventive services (AOR: 0.87) and more likely to receive restorative (AOR: 1.11) and oral surgery services (AOR: 1.23) [58]. In this study, periodontal care was more common in the urban area, which may indicate a similar trend, but surgical procedures need to be studied in more detail in the future.

### 4.6. Limitation of This Study

In this study, we used the NDB data to examine the provision of oral and dental care service delivery. However, there are two limitations. One is that the NDB does not include paper receipts from healthcare providers (3.6%) or patients receiving welfare payments due to poverty. Additionally, in the analysis, the economic indicators examined were prefectural averages and therefore do not accurately reflect an individual’s ability to pay. Moreover, the number of receipts was considered in this study and it was not possible to analyze all the costs incurred for tests and treatment per disease.

## 5. Conclusions

In this study, we investigated the provision of oral and dental health care services at the level of prefectures and secondary medical care areas using the National Database of Health Insurance Claims and Specific Health Checkups (NDB). The results showed that the total amount of oral care services provided tended to be higher in metropolitan areas, with bimodal peaks in children aged 5–9 and adults in their 70s. SCR for dental caries showed little difference nationally, but SCR for periodontal disease tended to be higher in prefectures including metropolitan areas. In the multiple regression model, the number of dentists and the per capita income of the prefectures influenced the SCR of total dental receipts. In the secondary healthcare areas, some of the depopulated areas were complemented by adjacent areas.

These results suggest that oral health care services in the national health insurance system are generally well provided in Japan, but may be affected by human resources and economic disparities, and that regional differences may occur in periodontal treatment. Although the use of social claim data appears to be limited in many countries, the accumulation of evidence in this area is necessary in the future to enlarge the coverage of public dental care in each country.

## Figures and Tables

**Table 1:** The prefectural characteristics in FY2016 and FY2018.

Fiscal Year	FY 2016				FY 2018			
Prefectural Characteristics	Mean	Std. Dev.	Min	Max	Mean	Std. Dev.	Min	Max
SCR of total number of receipts	94.53	10.28	71.24	118.52	94.30	9.98	70.49	116.87
Number of dentists ^§^	2160.7	2756.4	340	16,107	2165.5	2757.0	341	16,023
Number of dental hygienist	2634.7	2453.7	698	12,952	2821.9	2638.7	734	13,720
Number of dental technician	737.0	581.0	236	3013	733.4	594.7	230	3130
Number of dental laboratory	444.8	408.9	73	1846	446.9	407.1	78	1808
Number of dental clinics	1467.0	1855.0	257	10,658	1460.0	1854.0	260	10,672
Prefectural income (JY)	9,043,117	12,300,000	1,350,373	73,600,000	9,295,932	12,600,000	1,409,584	74,800,000
Prefectural population	2,700,697	2,742,348	569,554	13,600,000	2,690,280	2,766,906	560,397	13,800,000
(Per 1000 people)								
Dentists	0.73	0.13	0.55	1.18	0.74	0.13	0.55	1.16
Dental hygienist	1.06	0.22	0.67	1.60	1.13	0.23	0.73	1.68
Dental technician	0.32	0.10	0.16	0.61	0.3	0.10	0.15	0.62
Dental laboratory	0.17	0.03	0.10	0.25	0.17	0.03	0.10	0.25
Dental clinics	0.50	0.07	0.38	0.77	0.50	0.07	0.39	0.77
Prefectural income per capita ^Ψ^	2.92	0.48	2.37	5.40	3.03	0.47	2.47	5.41

FY: fiscal year, Std. Dev.: standard deviation, SCR: Standardized Claim Ratio, JY: Japanese yen, §: dentists in practice at healthcare facilities, ^Ψ^: million JY.

**Table 2:** Spearman’s correlations between the SCR of total dental receipts and prefectural characteristics in FY2018.

(Numbers Per 1000 People)	Dentists	Dental Hygienist	Dental Technician	Dental Clinics	Dental Laboratory	Income Per Capita ^Ψ^
SCR	0.522 ***	0.111	−0.419 *	0.501 ***	0.054	0.399 **
Dentists	1.0	0.241	−0.140	0.799 ***	0.376 **	0.104
Dental hygienist		1.0	0.399 *	0.2683 (*p* = 0.068)	0.242	−0.360 *
Dental technician			1.0	−0.267 (*p* = 0.069)	0.206	−0.281 (*p* = 0.056)
Dental clinics				1.0	0.357 *	0.138

^Ψ^: million Japanese yen, * *p* < 0.05, ** *p* < 0.01, *** *p* < 0.001.

**Table 3:** Multiple linear regression models as the SCR of total dental receipts an outcome variable in FY2018.

Model 1	(R^2^ = 0.4658, adj-R^2^ = 0.4285)				95% CI		
	Coef.	Std. Err.	t	*p*	low	high	vif
Dentists	32.82	9.30	3.53	0.001	14.07	51.57	1.28
Dental technician	−21.86	11.33	−1.93	0.06	−44.72	0.99	1.21
Prefectural income per capita	6.48	2.69	2.41	0.020	1.07	11.90	1.07
const.	57.75	9.53	6.06	<0.001	38.52	76.97	
Model 1_a	(R^2^ = 0.5107, adj-R^2^ = 0.4641)				95% CI		
	Coef.	Std. Err.	t	*p*	low	high	vif
Dentists	36.85	9.23	3.99	<0.001	18.22	55.49	1.27
Dental technician	−20.68	10.99	−1.88	0.067	−42.86	1.50	1.07
Prefectural income per capita	10.40	3.28	3.17	0.003	3.78	17.01	1.97
Dentist × Prefectural income per capita §	−19.52	9.94	−1.96	0.056	−39.59	0.54	2.03
const.	101.46	3.71	27.38	<0.001	93.98	108.94	
Model 2	(R^2^ = 0.3939, adj-R^2^ = 0.3516)				95% CI		
	Coef.	Std. Err.	t	*p*	low	high	vif
Dental clinics	57.03	21.36	2.67	0.011	13.97	100.10	1.43
Dental laboratory	13.12	39.32	0.33	0.74	−66.19	92.42	1.06
Prefectural income per capita	7.21	2.99	2.41	0.02	1.19	13.24	1.39
const.	41.94	11.28	3.72	0.001	19.18	64.69	
Model 2_a	(R^2^ = 0.4736, adj-R^2^ = 0.4234)				95% CI		
	Coef.	Std. Err.	t	*p*	low	high	vif
Dental clinics	85.91	23.17	3.71	0.001	39.16	132.67	1.89
Dental laboratory	−31.92	41.16	−0.78	0.442	−114.98	51.15	1.30
Prefectural income per capita	12.41	3.49	3.56	0.001	5.37	19.45	2.13
Clinics × Prefectural income per capita §	−50.09	19.87	3.56	0.016	−90.18	−9.99	2.98
const.	100.57	7.31	13.76	<0.001	85.82	76.97	
Model 3	(R^2^ = 0.5296, adj-R^2^ = 0.4813)				95% CI		
	Coef.	Std. Err.		*p*	low	high	vif
Dentists	21.89	9.99	2.19	0.034	1.74	42.04	1.54
Dental hygienist	14.26	6.06	2.35	0.023	2.03	26.49	1.69
Dental technician	−34.77	12.09	−2.88	0.006	−59.17	−10.38	1.31
Prefectural income per capita	8.96	2.78	3.22	0.002	3.35	14.56	1.52
const.	46.43	10.41	4.46	<0.001	25.43	67.44	
Model 3_a	(R^2^ = 0.5799, adj-R^2^ = 0.5287)				95% CI		
	Coef.	Std. Err.		*p*	low	high	vif
Dentists	25.28	9.74	2.60	0.013	5.62	44.95	1.60
Dental hygienist	15.29	5.88	2.60	0.013	3.41	27.16	1.74
Dental technician	−34.21	11.55	−2.96	0.005	−57.53	−10.89	1.35
Prefectural income per capita	13.26	3.27	4.06	<0.001	6.67	19.86	2.29
Dentists × Prefectural income per capita §	−20.22	9.33	−2.17	0.036	−39.06	−1.38	1.97
const.	88.57	6.05	14.63	<0.001	76.34	100.80	

FY: fiscal year, Coef: coefficient, Std. Err: standard error, CI: confidence interval, vif: variance inflation factor, SCR: Standardized Claim Ratio, JY: Japanese yen, adj-R2: adjusted R2, const: constant term, §: interaction term. All data were centralized to avoid multicollinearity. Dentists, dental hygienist, dental technicians, dental clinics, and dental laboratories are the number per 1000 people.

## Data Availability

The authors applied to access the original data of the Ministry of Health, Labour, and Welfare on 18 July 2019 according to NDB guideline [25].
